# Identification of regulatory elements flanking human *XIST *reveals species differences

**DOI:** 10.1186/1471-2199-11-20

**Published:** 2010-03-08

**Authors:** Samuel C Chang, Carolyn J Brown

**Affiliations:** 1 Department of Medical Genetics, Molecular Epigenetics Group, Life Sciences Institute, University of British Columbia 2350 Health Sciences Mall, Vancouver BC V6T 1Z3, Canada

## Abstract

**Background:**

The transcriptional silencing of one X chromosome in eutherians requires transcription of the long non-coding RNA gene, *XIST*. Many regulatory elements have been identified downstream of the mouse *Xist *gene, including the antisense *Tsix *gene. However, these elements do not show sequence conservation with humans, and the human *TSIX *gene shows critical differences from the mouse. Thus we have undertaken an unbiased identification of regulatory elements both downstream and upstream of the human *XIST *gene using DNase I hypersensitivity mapping.

**Results:**

Downstream of *XIST *a single DNase I hypersensitive site was identified in a mouse undifferentiated ES cell line containing an integration of the human *XIC *region. This site was not observed in somatic cells. Upstream of *XIST*, the distance to the flanking *JPX *gene is expanded in humans relative to mice, and we observe a hypersensitive site 65 kb upstream of *XIST*, in addition to hypersensitive sites near the *XIST *promoter. This -65 region has bi-directional promoter activity and shows sequence conservation in non-rodent eutheria.

**Conclusions:**

The lack of regulatory elements corresponding to human *TSIX *lends further support to the argument that *TSIX *is not a regulator of *XIST *in humans. The upstream hypersensitive sites we identify show sequence conservation with other eutheria, but not with mice. Therefore the regulation of *XIST *seems to be different between mice and man, and regulatory sequences upstream of *XIST *may be important regulators of *XIST *in non-rodent eutheria instead of *Tsix *which is critical for *Xist *regulation in rodents.

## Background

**Figure 1:**
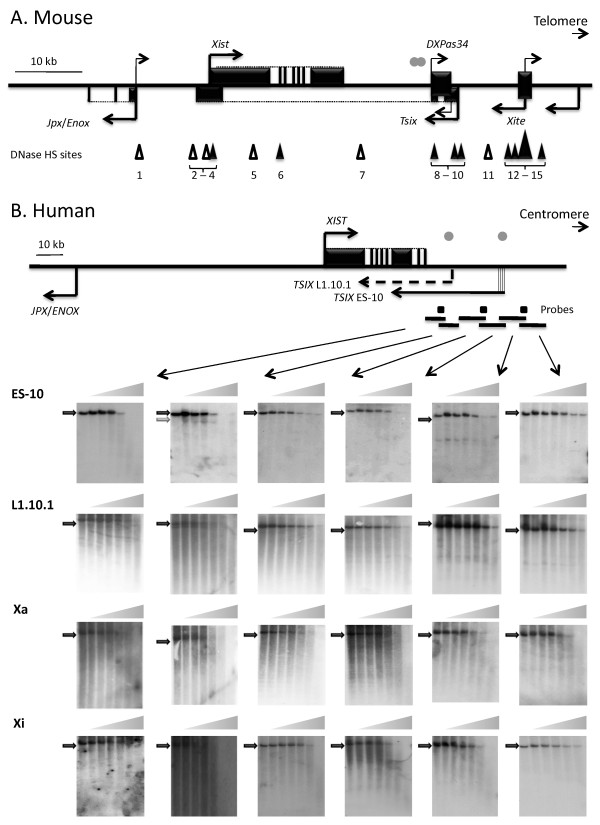
**Mapping DNase I hypersensitive sites downstream of *XIST***. A schematic of the mouse (A) and human (B) *Xist*/*XIST *genes and surrounding regulatory elements shows exons (black boxes) for *Xist*/*XIST *and other genes in the region (arrows indicate the direction of transcription). Grey dots show the location of conserved blocks between mouse and human [15], and triangles mark DNase I hypersensitive (DHS) sites (filled triangles - sites in undifferentiated cells; empty triangles - sites in differentiated cells; HS1, HS5 - HS7 [40], HS2 - HS4 [46,47], HS8 - HS15 [55]). DNA from nuclei of ES-10, L1.10.1 and Xa and Xi-containing hybrids exposed to DNaseI was digested with restriction enzymes (*Sca*I (10 kb); *Sap*I/*Blp*I (10.2 kb); *Nsi*I (11.1 kb); *Xmn*I (9.3 kb); *Nco*I (11.1 kb) and *Bam*HI (6.4 kb)) for Southern analysis. All experiments were carried out at least twice and a representative Southern blot is shown for each fragment including a lane with no DNase I (parental fragment shown as black arrow) followed by six lanes of increasing amount of DNase I treatment. One HS site (grey arrow), was detected in the *Sap*I/*Blp*I fragment of ES-10 cells, with a size of ~7 kb. No HS site was detected in the *Xmn*I fragment which encompasses the four previously reported transcription start sites of *TSIX *[32]. No HS site was detected in either the Xa or Xi hybrid downstream of *XIST*.

X-chromosome inactivation results in transcriptional silencing of one of the two X chromosomes in female mammals, thereby ensuring dosage equivalence of most X-linked genes between males and females. The X inactivation centre (*XIC*) is a single locus on the X chromosome that is required in *cis *for X-chromosome inactivation [1]. The *XIC *contains the X Inactive Specific Transcript (*XIST*/*Xist*) gene that produces a large (approximately 17 kb) noncoding RNA [2-4] that is necessary and sufficient to induce X inactivation [5-7]. X inactivation occurs very early in mammalian development, making analysis of the initial events challenging, particularly in humans. Most analyses have therefore been done in mouse, where ES cells not only provide the ability to generate targeted mutations, but also provide an *in vitro *system modelling X inactivation, as female ES cells undergo random X inactivation upon differentiation [8]. Detailed analyses of the genomic region downstream of the mouse *Xist *locus have revealed several *cis*-acting regulatory elements for *Xist *(see Figure 1A), including *Tsix *[9], *DXPas34 *and *Xite *[10].

The *Tsix *gene encodes an untranslated RNA antisense to *Xist *that is transcribed across the *Xist *locus, extending beyond the promoter of the sense strand [9,11]. A critical role for *Tsix *in regulating *Xist *expression was demonstrated by augmented *Tsix *expression resulting in inhibition of Xist accumulation [12] while deletion of the *Tsix *promoter resulted in primary non-random inactivation of the mutant X in females [13]. *DXPas34*, a 1.2 kb CG-rich region located 750 bp downstream of the *Tsix *major promoter, is critical for *Xist *and *Tsix *regulation, and shows bi-directional promoter activity [14,15]. *Xite*, named as the X-inactivation intergenic transcription element, was identified by DNase I hypersensitive mapping, and is also a site of bi-directional transcription, although the transcripts themselves are not necessary for *Xite *to promote *Tsix *persistence on the active X [10]. Homologous pairing of a region downstream of *Xist*, encompassing *Tsix*, *DXPas34 *and *Xite*, is necessary for the initiation of X inactivation [16,17]. This pairing can be recapitulated by sub-fragments which contain a high density of CTCF sites, and CTCF as well as transcription is essential for the establishment of pairing [16].

While the mouse system provides an excellent framework from which we can understand the process of X inactivation, how human fits into this framework is not clear. *XIST *and *Xist *share sequence homology [3,4] and are each sufficient to initiate silencing [18,19]; however, there are substantial differences between the two species in other aspects of X-chromosome inactivation. Significantly, X inactivation is imprinted in mouse extraembryonic tissues [20] but not in humans (reviewed in [21]) and *Tsix *is very different from *TSIX *in patterns of expression and extent of transcription across the sense strand [22-24]. Human *TSIX *has been observed in embryoid bodies and a human embryonal carcinoma cell line, but also in chorionic villus cells [22,24]. *TSIX *is also expressed in mouse ES-10 cells carrying a multi-copy integration of a 480 kb *XIST*-containing human YAC transgene [25] and in L1.10.1, a clone of a somatic male cell line transfected with a human *XIST*-containing PAC, which contains at least 50 kb of flanking genomic DNA [26]. While these cells did not show identical initiation sites for the TSIX transcript, in all cases the TSIX transcript truncated well before the 5' end of *XIST*. As human ES cells have been variable in their X inactivation status (*e.g. *[27]), mouse ES cells with a human transgene remain one of the best models for human X inactivation. Multi-copy integrations of the 480-kb transgene containing human *XIST *display partial X inactivation center function upon *in vitro *differentiation of male mouse embryonic stem cells, including activation in some cells of the endogenous mouse *Xist *locus [28,29].

Given the importance of *XIST *for X inactivation, it is perhaps surprising that the X inactivation center region shows little sequence conservation surrounding *XIST *between mouse and human [30,31]. In addition to *XIST*, the region contains several conserved genes and a number of non-coding RNAs and pseudogenes. Downstream of *Xist *the closest gene is the testes-specific *Tsx *gene in mouse, however *TSX *is a pseudogene in human [32]. The closest gene upstream of *XIST *is the *JPX *(also known as *ENOX*) non-coding RNA gene. Interestingly, the region between *JPX *and *XIST *is ~90 kb in human, which is approximately 9 times larger than that of mouse. Thus rearrangements downstream of *XIST *where the mouse regulatory elements are found may have been compensated for by regulatory regions upstream of the gene. To identify such regulatory elements in the absence of substantial sequence conservation we have used DNase I hypersensitive site mapping as an unbiased approach to identify *cis*-acting regulatory elements in humans. Genomic regions hypersensitive to DNase I digestion (DHS sites) have been shown to harbour *cis*-regulatory elements critical for gene regulation [33], and both of the mouse *Xist *regulatory regions *DXPas34 *and *Xite *show DNase I hypersensitivity [12]. Here, we report the identification of three previously unknown hypersensitive sites surrounding the human *XIST *locus.

## Results

### Identification of DHS sites 3' to human *XIST*

The critical timing for *XIST *regulation is early in development and thus for mapping of DHS sites 3' to human *XIST *we used the mouse undifferentiated embryonic stem cell line ES-10 which contains a human *XIST *transgene. We also examined the L1.10.1 cell line which is a somatic male HT1080 cell line transfected with a PAC containing the *XIST *region, and which expresses *XIST *as well as *TSIX*. Additionally, we examined mouse/human somatic cell hybrids containing either the human active (Xa) or inactive (Xi) chromosome, which provide a unique opportunity to compare the chromatin structure between the Xi and Xa independent of each other without requiring allele-specific detection. We generated three probes downstream of *XIST *that were able to detect six overlapping restriction fragments allowing the analysis of a 43 kb region (see Figure 1B). Only one DHS site, located approximately 12 - 13 kb downstream of *XIST*, was identified in the ES-10 cells. This DHS site does not correspond to the cluster of four *TSIX *transcription start sites described in these cells, which are located approximately 14 kb further from the 3' end of *XIST *[25]. The DHS site is close to the antisense transcription start in L1.10.1 cells. Interestingly, however, the DHS site was not found in L1.10.1 (Figure 1B), nor were any others, despite previous detection of antisense transcript both with RT-PCR and FISH in this cell line [24]. We confirmed the ongoing presence of antisense transcript in the L1.10.1 cells used for DHS mapping by RT-PCR (data not shown). DHS mapping in both Xi and Xa hybrids did not show the presence of any DHS site downstream of *XIST*, including at the region identified to contain a DHS in ES-10 cells (Figure 1B). It thus appears that, unlike the situation in mouse where there are both developmental-specific and constitutive DHS sites downstream of *Xist*, in humans there is only a single developmental-specific DHS site downstream of *XIST*.

### Identification of DHS sites 5' to human *XIST*

The lack of putative regulatory elements 3' to *XIST*, where many of the mouse transcriptional regulatory sites are located, led us to examine the region 5' to *XIST *which is larger in humans than it is in mouse [30]. We generated three probes to examine the region upstream of human *XIST *for DHS sites. Almost 80% of the just over 90 kb region upstream of *XIST *is comprised of repetitive elements, predominantly LINE1 (39.5%) and ALU (27.3%) as identified by repeatmasker (http://www.repeatmasker.org/[34]). This high repeat content precluded examination of the entire region and the restriction fragments assessed by the three probes interrogate a total of 39 kb in the 90 kb region.

**Figure 2:**
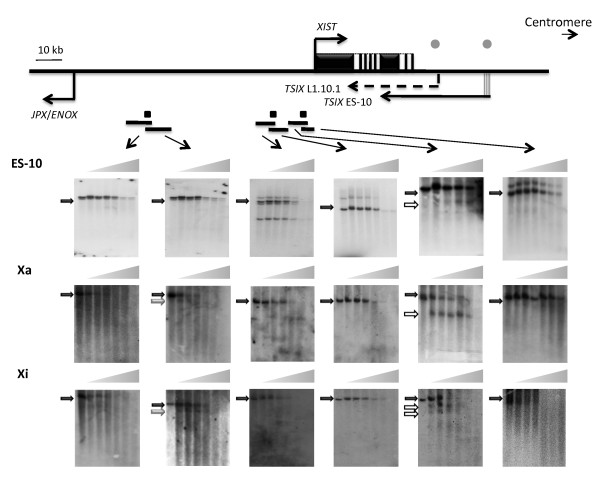
**DNase I hypersensitivity mapping upstream of *XIST***. Below the schematic of the *XIST *region (see legend in Figure 1) are Southern blots of DNA from ES-10, an Xa hybrid, and an Xi hybrid. Small rectangular boxes are probes for Southern blotting of overlapping restriction fragments (from left to right: *Pst*I (13.1 kb); *Bsm*I (10.3 kb); *Nde*I (7.2 kb); *Sap*I (6.7 kb); *Spe*I (6.3 kb); *Sca*I (3.8 kb)). All experiments were carried out at least twice. One HS site, highlighted by the open arrow, was detected just upstream of the transcriptional start site, within the *Spe*I fragment, at somewhat variable positions in the three cell lines. Another HS site was found within the *Bsm*I fragment approximately 65 kb upstream of the *XIST *transcription start in both Xa and Xi hybrids (grey arrow). No other DNase I hypersensitive sites were observed.

We again performed Southern analysis with DNA isolated after increasing DNase I treatment from cells of ES-10, an Xa-containing, and an Xi-containing somatic cell hybrid (Figure 2). The presence of multiple bands for proximal but not distal probes in ES-10 can be explained by the individual copies of the *XIST *transgene present in the multi-copy integration transgenes not containing the same amount of DNA sequence flanking the *XIST *locus. We observed one or more DHS site(s) immediately upstream of *XIST *in ES-10, as well as in the Xa-containing hybrid where *XIST *is silenced. These sites were observed variably in Xi-containing hybrids where *XIST *continues to be expressed (Figure 2). While no other hypersensitive sites were detected upstream of *XIST *in ES-10, one DHS site was found approximately 65 kb upstream of the *XIST *transcription start on both the active and inactive X chromosomes in somatic hybrids (Figure 2).

**Figure 3:**
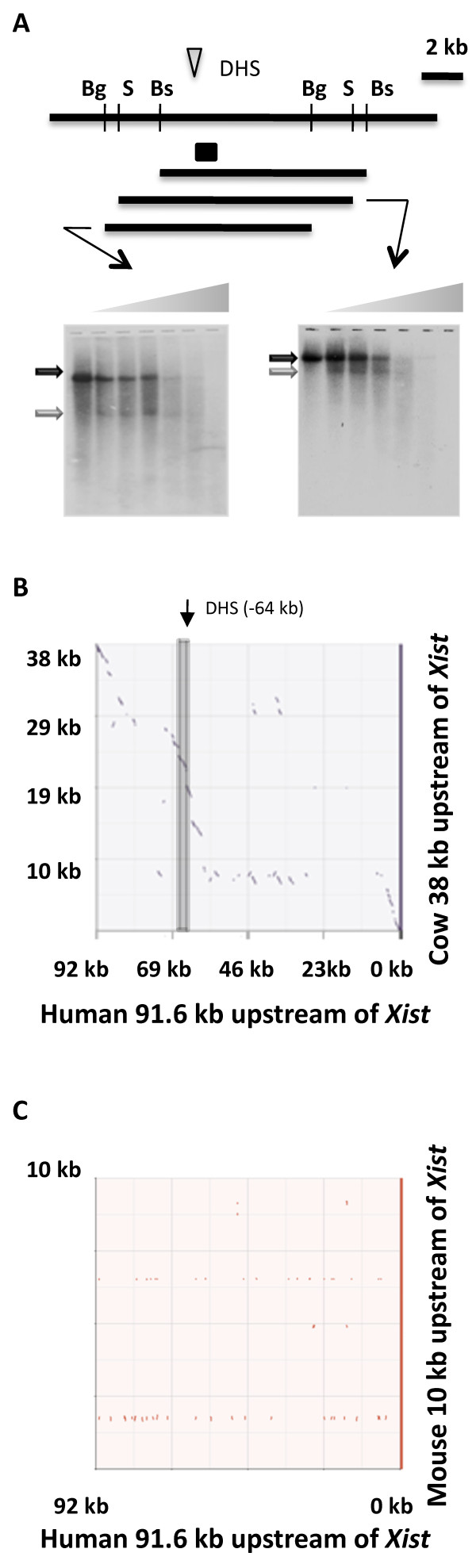
**Localization of the -65 DNase I hypersensitive site upstream of *XIST***. A. Using an Xa hybrid to limit background from cross-hybridization to human material, two additional restriction digests were performed on different DNA preparations after increasing DNase I treatments to refine the location of the -65 site. The *Bgl*II fragment is 9.3 kb and the fragment resulting from HS and restriction digestion is 5 kb (grey arrow, left panel). The *Sca*I fragment is 11.8 kb and the fragment resulting from HS and restriction digestion is 8 kb (grey arrow, right panel). Dot-plot analyses http://mulan.dcode.org/ showing comparison of sequence upstream of *XIST *between cow (38 kb) and human (92 kb) (panel B) and mouse (10 kb) and human (92 kb) (panel C).

The location of the -65 DHS site was refined by repeating the DHS mapping with two other restriction enzyme digests for the Xa-containing hybrid (Figure 3A), thereby refining the location of the site to between 73,053,323 and 73,053,866 (hg18). A dot-plot sequence comparison of human to cow, and human to mouse sequences showed that within the region approximately 60 kb upstream of *XIST *there is a region of approximately 10 kb that is relatively conserved between human and cow, but not mouse (Figure 3B and 3C). This region is also conserved in dog (data not shown). The novel -65 DHS site identified with both the Xa and Xi in somatic hybrids is located within this conserved region.

**Figure 4:**
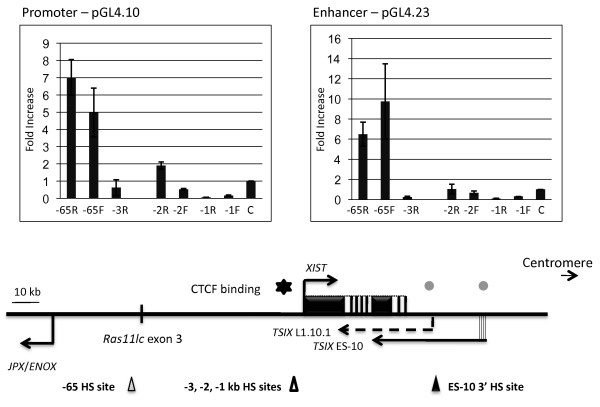
**Dual luciferase reporter assays examining promoter and enhancer activities for DNA fragments containing HS site**. The pGL4.10 vector, which contains the promoterless synthetic firefly *luc2*, was used in the promoter assay (left). The pGL4.23 vector, which contains the synthetic firefly *luc2 *driven by a minimal promoter, was used in the enhancer assay (right). The histograms show a summary of the ratio of luciferase activity (adjusted by dividing the firefly luciferase with the control *Renilla *luciferase) for each insert (from the HS sites shown in lower panel) relative to the luciferase activity for pGL4.10 or pGL4.23. Each fragment was tested in triplicate and experiments were carried out three times independently. Error bars represent the standard deviations of three trials. While fragments upstream of the *XIST *promoter containing HS sites showed background luciferase activity, fragment -65 displayed five fold and seven fold increases in promoter activity and ten fold and six fold increases in enhancer activity in the *XIST *and antisense orientation, respectively.

To determine the biological relevance of this conserved sequence that results in a DHS in somatic cells we cloned several regions of *XIST *into the pGL4 series of plasmids to assay promoter and enhancer activity by monitoring luciferase reporter activity after transient transfection into HT1080 somatic cells (Figure 4). We cloned three ~700 bp regions of the *XIST *promoter region (named -3, -2, -1) as well as a 1,087 bp region at the -65 DHS site in both orientations. There was a notable orientation bias for regions around the *XIST *promoter, and in fact -3 could not be cloned in one orientation. As pGL4.10 lacks a minimal promoter, assaying luciferase activity monitors promoter activity; while pGL4.23 contains a minimal promoter and therefore monitors enhancer activity. In both assays the -65 region showed significant activity, when cloned in both orientations. Thus, like many of the mouse regulatory regions, the -65 DHS seems to have bi-directional promoter and enhancer activity.

## Discussion

X-chromosome inactivation in both humans and mice requires the presence in *cis *of the X-inactivation center, and the *XIST/Xist *gene contained therein. Interestingly, the *XIST *gene appears to have evolved in eutherians from a protein-coding gene, *Lnx3 *[31], and while critical regions such as the 5' A repeats required for silencing are conserved [19,35], other regions of *XIST *are more variable amongst the eutheria [36]. Extensive studies in mouse models have defined a wide variety of regulatory elements for *Xist *including *Tsix*, *DXPas34 *and *Xite*. These elements are all located 3' to *Xist*, between *Xist *and the adjacent testes-specific *Tsx *gene. In humans *TSX *is a non-expressed pseudogene, and the blocks of sequence conservation previously reported between humans and mice 3' to *XIST *are ancestral *TSX *exons [31,32]. The human *TSIX *region lacks an equivalent to the mouse CpG island that was shown to be essential for function of *Tsix *[13,32,37]. Other significant differences between mouse and human *Tsix*/*TSIX *include a lower level of human *TSIX *transcription and termination of human *TSIX *prior to the *XIST *promoter [24,32], while antisense transcription across the mouse *Xist *promoter region is necessary for antisense function [38]. In addition, Migeon et al. (2002), using RNA FISH for cellular localization of transcripts, showed that human *TSIX *transcripts are co-expressed with *XIST *from the inactive X throughout human embryonic development, suggesting that this antisense is unable to repress *XIST *[22] leading to the argument that *Tsix *regulation of *Xist *may be specific to mouse [39].

Upstream of *XIST/Xist *the adjacent gene is *JPX/ENOX*, which is conserved in humans and mice [30], although the distance to the *JPX *CpG island and first exon is larger in humans (~90 kb) than in mice (less than 10 kb) [30]. *JPX *is a non-coding RNA gene including different repetitive elements in different species, and the promoter of the mouse *Jpx *gene has been shown to interact with the *Xist *promoter in undifferentiated ES cells [40]. No sequence conservation between humans and mice is found in the region between *Jpx *and *Xist*; however, there is conservation between humans and cows (and also dogs, data not shown), including an exon of the *Rasl11c *pseudogene [31].

The lack of sequence conservation between human and mouse in the region flanking *XIST/Xist*, as well as the differences in the TSIX/Tsix transcript, led us to undertake this study to identify potential regulatory elements for *XIST *by using DHS mapping both downstream and upstream of *XIST*. Downstream of *XIST *in the region where most regulatory elements are observed for mouse *Xist *we find only a single DHS, and this is only observed in undifferentiated mouse ES cells containing a human *XIC *transgene. While this site mapped close to one of the previously described human *TSIX *transcription start sites, the presence of the DHS did not correlate with *TSIX *transcription, and thus we consider it more likely that this DHS is reflective of a developmental event not regulating antisense transcription. As previously reported there is no substantial sequence conservation between cow, human and mouse downstream of *XIST *[24,30], and dog also fails to show homologous regions (data not shown). In this region downstream of *XIST *the human sequence is enriched for LTR class repetitive elements, while mouse and dog are enriched in LINE elements. It has been demonstrated that in addition to a *cis*-regulatory role in *XIST *regulation, the mouse *Tsix *and *Xite *regulatory elements are also involved in a *trans*-regulation involving a transitory pairing of homologous X chromosomes proposed to establish the mutually exclusive choice of the future active and inactive X chromosomes in females [41]. As the human *XIC *transgene in the ES-10 cells studied is capable of inducing expression of the single *Xist *gene in these male ES cells [29], it is plausible that the DHS identified in the transgene 3' to *XIST *is involved in such trans-interactions. There is now genome-wide mapping of DNase I hypersensitive sites [42] as well as histone modifications and CTCF binding sites, which often mark promoters and enhancers [43,44] in a number of cultured cell lines or human CD4+ cells. The genome-wide mapping of CTCF sites in somatic cells did not identify any enrichment in the region of this 3' DHS. However, we also did not observe this DHS in somatic cells. Enhancer sequences have been reported to show cell-type specific patterns [45], so this region may contain an enhancer specific to undifferentiated cells.

We found evidence for DHS sites near the transcriptional start site of *XIST *in both somatic and ES-10 cells. *XIST *is expressed in the ES-10 cells prior to differentiation, however it is not expressed in the Xa-containing hybrid cells, thus these sites are observed independent of *XIST *expression. We did not refine the localization of these sites, as they likely correspond to the minimal *XIST *promoter and regulatory elements. The minimal mouse promoter has been shown to have a cluster of DHS sites [46,47]. In agreement with the previously defined human minimal promoter [48] we detected strong promoter activity in our transient luciferase assay using 1 kb of DNA surrounding the *XIST *transcription start site (data not shown). Although a CTCF binding site has previously been defined at -43 bp of the *XIST *promoter [49], genome-wide mapping in CD4^+ ^T cells only identified CTCF binding further upstream [43]. This would correspond to our fragment 3 which showed limited promoter or enhancer activity in the 'reverse' orientation, but was unable to be cloned in the 'forward' orientation where it would be aligned with the test promoter in the same orientation it is aligned to *XIST *in the human genome. Genome-wide mapping of DHS sites identified a DHS site approximately three kb upstream of the promoter, in the vicinity of the CTCF site, as well as sites further within *XIST *that our analysis would not have detected [42]. Thus it appears that there is a regulatory element for human *XIST *~three kb upstream of the *XIST *promoter which includes CTCF binding sequences.

Further 5' to *XIST *we find a DHS site in a region sharing sequence conservation with cow and dog (data not shown). A DHS site in this region can also be observed in the genome-wide mapping in CD4^+ ^T cells, and furthermore genome-wide H3K4me1 enrichment, which is characteristic of enhancers, flanks the site [45]. We refined the location of the DHS to between 73,053,323 and 73,053,866, while the DHS site identified by the global mapping is slightly proximal at 73,053,237- 73,053,138. This might reflect subtle discrepancies in mapping, or between cell types. The region cloned for subsequent enhancer and promoter activity analysis was 73,054,787 -73,053,643 and would contain the H3K4me1 marked regions [45] and the majority of the region to which our DHS site was mapped, but be just upstream of the DHS site mapped in CD4+ cells [42].

Overall we find fewer regulatory elements in human than have been identified in mice. This could be due to higher repetitive element content in human which made analyzing the whole region challenging. Furthermore, the repetitive elements themselves might harbour regulatory elements. Indeed, the *TSIX *transcription starts mapped by Migeon *et al*. were to MER58B, AluY and L2 class repetitive elements [32], and conservation of repetitive elements between the mouse *Tsix *transcription start and humans was noted by Cohen et al. [15]. While acquisition of repetitive elements may have led to an expansion of the *XIC *region in humans compared to mice, the conservation upstream of *XIST *between humans and other eutheria, including homology to an exon of the *Rasl *gene of chicken [31] suggests that this region was likely lost in rodents.

While many *XIST *regulatory elements do not appear to be conserved between humans and mice, many of the basic events required for X-chromosome inactivation must be conserved (reviewed in [21]). The 3' DHS site might demarcate a developmental-specific regulatory region that participates in the *trans *pairing interactions involved in initiation [17,41]. It has been proposed that a critical function of *TSIX *is to partition chromatin domains in the *XIC *[50]. Perhaps the 5' regulatory regions we have identified are capable of recapitulating such a function in humans. However we did not observe a consistent difference between the active and inactive X chromosomes for these DHS sites, and genome-wide chromatin mapping in somatic cells does not show evidence for a chromatin domain ending at the -65 DHS. Ultimately testing whether human X inactivation involves regulatory processes related to those detailed in mouse will require the challenging investigation of human *XIST *expression during early development. It has been shown that X chromosome inactivation is initiated in human preimplantation embryos [51]; however, human ES cells have shown considerable variability (e.g. [27]), making mouse ES cells with human *XIST *transgenes one of the best current models to study regulation of human *XIST*.

## Conclusions

DNase I hypersensitivity mapping around the human *XIST *gene has identified fewer candidate regulatory regions than are observed flanking mouse *Xist*. In particular, 3' to *XIST *only a single, developmental-specific DHS site was observed in undifferentiated mouse ES cells with an integration of the human *XIST *domain. 5' to *XIST *a DHS site was identified in a region of sequence conservation amongst non-rodent eutheria. This region showed bi-directional promoter and enhancer activity. The lack of conservation of regulatory elements for *XIST *lends support to previous conjectures that human *TSIX *is no longer a functional regulator of *XIST*.

## Methods

### Tissue Culture & Cell Lines

ES-10 cells, a derivative of J1 male mouse embryonic stem cells with a 480-kb human *XIC *transgene, were generously provided by Dr. B. Migeon and maintained as described [22]. The L1.10.1 transgenic derivative of human male fibrosarcoma cell line HT-1080 was grown as described [19]. Mouse-human somatic cell hybrids t11-4Aaz5 (containing a human Xi as well as six human autosomes in addition to mouse chromosomes) and t60-12 (containing a human Xa) were maintained as described [52].

### DNase I Hypersensitivity Mapping

The preparation of nuclei and the DNase I digest were as described [53]. Briefly, cells were harvested and washed twice in ice-cold PBS, then resuspended at 1 × 10^7 ^cells/ml in 10 ml ice-cold sucrose-triton, swelled on ice for 15 minutes, and then homogenized 10 times in a Dounce homogenizer with a B pestle (7 ml, Wheaton). Homogenized cells were transferred to a 15-ml falcon tube and spun at 1,200 rpm for 15 min at 4°C to recover nuclei which were then resuspended in 1.5 ml of ice cold buffer (50 mM Tris-Cl pH7.9, 100 mM NaCl, 3 mM MgCl_2_, 1 mM dithiothreitol, 0.2 mM phenymethylsulfonyl fluoride) and aliquoted into seven 1.5 ml eppendorf tubes (200 μl each). Nuclei were digested with an increasing amount of DNase I (10 U/μl, RNase free, Roche) (i.e. 1/128, 1/64, 1/32, 1/16, 1/8, 1/4 U/μl) at 37°C for 20 minutes. The digestions were stopped and DNA extracted by adding 1 ml DNazol (Invitrogen) following the manufacturer's protocol. DNase I treated DNA was digested with restriction enzymes according to the manufacturer's recommendations and analyzed by Southern blotting with random-primed P^32^-labelled probes generated by PCR with primers listed in Table 1[53]. All analyses were repeated at least twice. As the cell lines contain variable proportions of the human genome they showed differences in cross-hybridization. In order to be identified as a DHS site a band could not be visible in the undigested lane and needed to be replicated.

**Table 1:** Primers for probe generation

Symbol	Size	Name	Sequence (5' to 3')
36	921 bp	IP368F	CTTGCTCACCAATTGACTCGTAAG
		IP359R	GAGGACGTGTCAAGAAGACACTAGG

45	874 bp	IP453F	CATGGGAAAGCAGCAGACTTCT
		IP444R	GGGCCTGAATGTGAGCATAGAT

101	1190 bp	IP1011F	GAATAGCTCAACTGCCAGTGTTACT
		IP1000R	GGTCCTCAATGTCCTTTACAAAGC

86	1041 bp	U862F	TGGAGTCCAGTCGTTGTGCT
		U873R	ATAATCTTGCTACTGAAGGGGCT

105	1209 bp	U1056F	TGCTTGAAGGGTTTACTGCTGTC
		U1068R	CTATACAATGCTCCTGTGATTCTAGTGC

117	1140 bp	U1179F	CTTCTGCACTCTGCTAAAGTTCTGAC
		U1190R	TCTGTGACTTGGCAAGCCTTC

### Sequence Comparisons, Plasmid Construction and Luciferase Assay

For the dot-plot analyses, we used Mulan (http://mulan.dcode.org/[54]). Five DNA fragments of interest were generated by PCR amplification of human genomic DNA from GM01416 for 35 cycles using primers listed in Table 2. PCR fragments were first cloned into pGEM^®^-T Easy vector (Promega) via TA cloning prior to insertion into pGL4.10 and pGL4.23 reporter vectors (Promega) upstream of the firefly luciferase gene. The identities of pGL4 clones were confirmed by restriction enzyme digestion and partial sequence analysis. Transient transfection was performed in 24-well plates with 80% confluent HT1080 cells. 0.8 μg of firefly luciferase plasmid (pGL4, Promega) and 80 ng of the *Renilla *luciferase plasmid (Promega) were co-transfected into cells using 2 μl Lipofectamine™ 2000 (Invitrogen). For each transfection assay, the pGL4.13 vector, which contains a promoter/enhancer element, was used as positive control and the pGL4.10 vector, which contains neither promoter nor enhancer, was used as negative control. Transfection of the pGL4.23 vector with a basal promoter also served as a control. After 24 h, cell lysates were prepared from each transfected culture and were analyzed in a 96-well plate luminometer (Perkin Elmer Wallace) according to manufacturer's protocol in the Dual Luciferase Kit (Promega). Each fragment was tested in triplicate, and each experiment was repeated at least three times. To control for transfection efficiency, the ratio of firefly luciferase signal to *Renilla *luciferase signal was calculated for each transfected sample.

**Table 2:** Primers for cloning

Fragment Content	Name	Sequence (5' to 3')
*XIST *promoter, positive control	0 F	TCGAGCTCCTTGCTCACCAATTGACTCGTAAG
	0 R	CGGGTACCGAGGACGTGTCAAGAAGACACTAGG

1 kb upstream of *XIST *promoter	-1 kb F	TCGAGCTCCATTTCCACACTTGTAGAAACTTCTAGTAG
	-1 kb R	CGGGTACCCTTACGAGTCAATTGGTGAGCAAG

2 kb upstream of *XIST *promoter	-2 kb F	TCGAGCTCGAGCCAAGCAGTAGTGAAGGTGA
	-2 kb R	CGGGTACCGGTTGTCCTGGGTTTCTGTGA

3 kb upstream of *XIST *promoter	-3 kb F	TCGAGCTCCCCCGTGTTCTCTTTTGATAAACTAG
	-3 kb R	CGGGTACCTCACCTTCACTACTGCTTGGCTC

65 kb upstream of *XIST*, covers HS 101	-65 kb F	TCGAGCTCGTGGAGTACCCTTTCTATCACAACT
	-65 kb R	CGGGTACCTGGCTTGACTTCTAGGGTAAAGA

* underlined is genomic sequence from NCBI; not underlined is adaptor (i.e. *Sac*I &*Kpn*I sites)

## Authors' contributions

SC carried out the molecular studies and drafted the manuscript. CB participated in the design of the study and revised the manuscript. Both authors read and approved the final manuscript.
